# Phenotypic flexibility in background-mediated color change in sticklebacks

**DOI:** 10.1093/beheco/araa041

**Published:** 2020-05-06

**Authors:** Petter Tibblin, Marcus Hall, P Andreas Svensson, Juha Merilä, Anders Forsman

**Affiliations:** 1 Ecology and Evolution in Microbial Model Systems, EEMiS, Department of Biology and Environmental Science, Linnaeus University, Kalmar, Sweden; 2 Ecological Genetics Research Unit, Organismal and Evolutionary Biology Research Program, Faculty of Biological and Environmental Sciences, University of Helsinki, Helsinki, Finland

**Keywords:** background matching, coloration, Gasterosteus, phenotypic plasticity, Pungitius

## Abstract

Phenotypic flexibility may incur a selective advantage in changing and heterogeneous environments, and is increasingly recognized as an integral aspect of organismal adaptation. Despite the widespread occurrence and potential importance of rapid and reversible background-mediated color change for predator avoidance, knowledge gaps remain regarding its adaptive value, repeatability within individuals, phenotypic correlates, and whether its expression is context dependent. We used manipulative experiments to investigate these issues in two fish species, the three-spined stickleback (*Gasterosteus aculeatus*) and nine-spined stickleback (*Pungitius pungitius*). We sequentially exposed individuals to dark and light visual background treatments, quantified color change from video recordings, and examined associations of color change with phenotypic dimensions that can influence the outcome of predator-prey interactions. *G. aculeatus* expressed a greater degree of color change compared to *P. pungitius*. In *G. aculeatus*, the color change response was repeatable within individuals. Moreover, the color change response was independent of body size but affected by sex and boldness, with males and bolder individuals changing less. Infection by the parasite *Schistocephalus solidus* did not affect the degree of color change, but it did modulate its association with sex and boldness. *G. aculeatus* adjusted the expression of color change in response to predation risk, with enhanced color change expression in individuals exposed to either simulated attacks, or olfactory cues from a natural predator. These results provide novel evidence on repeatability, correlated traits, and context dependence in the color change response and highlight how a suite of factors can contribute to individual variation in phenotypic flexibility.

## Teaser

Many organisms change their coloration rapidly as a response to visual backgrounds to avoid detection from predators. The capacity to change coloration varies not only between species but also among individuals within species. However, the sources of individual variation in color changing behavior are poorly understood. By studying sticklebacks, we show that color changing behavior was influenced by sex, boldness, and parasite infection. Moreover, individuals also changed their color to a higher degree when at risk of predation.

## INTRODUCTION

Phenotypic flexibility, also known as reversible phenotypic plasticity, is taxonomically widespread (Piersma and Drent 2003; Dingemanse et al. 2010). For long considered primarily as a nuisance in evolutionary biology, phenotypic flexibility and developmental plasticity are now recognized as integral parts of organismal adaptation to their environments (Pigliucci et al. 2006; Charmantier et al. 2008; Pfennig et al. 2010; Ghalambor et al. 2015; Merilä 2015; Edelaar and Bolnick 2019). For instance, theoretical and empirical work suggest that flexibility in behavior, physiology, morphology, and life history may incur a selective advantage in rapidly changing or fine-grained unpredictable, heterogeneous, and fluctuating environments (Piersma and Drent 2003; Lande 2014; Beaman et al. 2016; Forsman and Wennersten 2016; Tibblin et al. 2016). Moreover, the significance of flexibility (and plasticity) has been further emphasized by its key role in predicting whether organisms can cope with environmental heterogeneity and respond to the global warming and associated anthropogenic impacts (Charmantier et al. 2008; Seebacher et al. 2015).

Interspecific and interpopulation comparisons of phenotypic flexibility have advanced our understanding of the adaptive genetic basis of phenotypic flexibility and revealed that it may increase the success of species and populations in spatiotemporally variable environments (Sol and Lefebvre 2000; Phillimore et al. 2016). However, despite its potential evolutionary importance, the phenotypic correlates and sources of variation in phenotypic flexibility among individuals within populations have received little attention (Dingemanse and Wolf 2013; Forsman 2015; Beaman et al. 2016). For instance, in contrast to the extensive evidence of genotype-by-environment interactions regarding developmental plasticity, few studies have investigated whether variation among individuals in the expression of rapid phenotypic flexibility has a heritable component (Forsman 2015). In this context, measures of repeatability can be used to quantify the degree of within-individual variation in flexibility relative to the magnitude of variation seen among individuals (Martin and Réale 2008; Bell et al. 2009). Such repeatability estimates may be thought of as representing upper bounds of heritability, and thus provide a rough indication of evolvability of phenotypic flexibility (Boake 1989). Surprisingly little is also known about context dependence of individual expression of phenotypic flexibility. For example, the threat-sensitivity hypothesis posits that an individual’s flexible response should be modulated by predation risk, which would emphasize the adaptive value of the response, but this prediction has rarely been evaluated in flexible traits that are capable for rapid phenotypic responses. It also remains unclear whether and how such modulated responses are related to the individual’s “baseline” capacity for flexibility in the absence of predators (Helfman 1989; Edelaar et al. 2017).

A textbook example of phenotypic flexibility is the ability of some organisms to rapidly change their color to match their environment to avoid predation through camouflage (Merilaita et al. 1999; Leclercq et al. 2010; Sköld et al. 2013; Stevens 2016). Such physiological color change is a plastic and reversible response that manifests within seconds to hours (Stuart-Fox and Moussalli 2009; Sköld et al. 2013; Stevens 2016). Well-known examples include cephalopods (Hanlon et al. 2009) and fiddler crabs (Stevens 2016), but rapid color change is taxonomically widespread (Stuart-Fox and Moussalli 2009). For example, it is known that the dorsal area of fishes can become darker when exposed to dark visual backgrounds, presumably to reduce the risk of being detected by predators (Clarke and Schluter 2011; Sowersby et al. 2015; Kelley et al. 2016). The importance of protective animal coloration is undisputable, yet the sources of variation in color change ability are poorly understood. For instance, little is known on whether and how color changing behavior is associated with other phenotypic dimensions that affect the outcome of predator–prey interactions, such as sex, body size, health status, and boldness (Brönmark and Miner 1992; Forsman and Appelqvist 1999; Stuart-Fox and Moussalli 2009; Ahlgren et al. 2015; Stevens 2016; Duarte et al. 2017; Karpestam et al. 2018). With regards to health status, there are examples of parasites with complex life cycles that influence the behavior of their hosts in ways that increases the latter’s susceptibility to predation, thereby increasing the likelihood that the parasite is being passed on to later-sequence host species where they can complete development (Barber et al. 2000, 2004; Berdoy et al. 2000). Such parasite mediated increased susceptibility to predation may reflect an adaptive (from the parasites point of view) manipulation of host behavior or a byproduct of physiological stress (Poulin 1994). Associations between animal coloration and susceptibility to parasite infection may also arise due to developmental links and shared structural components involved in pigmentation and immune defense (Andersson 2001; True 2003; Protas and Patel 2008). All these proposed mechanisms predict that infected individuals should express a color changing behavior that is less efficient in terms of background matching, but this remains little explored (but see Seppälä et al. 2005).

Here, we explored individual variation in color change in two closely related fish species, the three-spined stickleback (*Gasterosteus aculeatus*) and the nine-spined stickleback (*Pungitius pungitius*). These small, mesopredatory, and widespread fish species are important model organisms in ecology and evolution (Gibson 2005; Merilä 2013). They have also contributed to substantial advances in our understanding of the adaptive value of phenotypic plasticity (Svanbäck and Schluter 2012; Foster et al. 2015; Dingemanse et al. 2020). *G. aculeatus* has also been in the center of considerable attention regarding its coloration: bright nuptial colors of males (Milinski and Bakker 1990; Rick and Bakker 2008; Lindström et al. 2009; Bolnick et al. 2015; Brock et al. 2017), color variation among populations (Greenwood et al. 2011, 2012), and identification of candidate genes underlying variation in pigmentation (Berman et al. 2009; Greenwood et al. 2011) are all topics that makes the species an important model system. Moreover, it has been demonstrated that *G. aculeatus* can rapidly change their coloration (darkness) in response to a change in the visual background, primarily by adjusting the darkness of their dorsal skin, and that this ability varies between ecotypes, indicating an adaptive basis for this variation (Clarke and Schluter 2011).

In this study, we report on the results of two manipulative experiments that were performed to investigate sources of individual variation in the color change behavior of *G. aculeatus* and *P. pungitius.* In the first experiment, we exposed wild-captured subjects originating from the Baltic Sea to both dark and light visual backgrounds under controlled laboratory settings in the absence of predatory cues. Using data from this experiment, we first evaluated whether the expression of color change differed between the two species (viz. *G. aculeatus* and *P. pungitius*). Although these species are ecological similar in many aspects, *G. aculeatus* and *P. pungitius* vary in spatiotemporal distribution, with the former having a distinct migratory behavior alternating between offshore pelagic and coastal littoral habitats and thus exposed to differences in predator regimes and visual context across its life cycle, whereas *P. pungitius* is chiefly sedentary in the coastal habitat (Bergström et al. 2015). Next, we investigated sources of individual variation and condition dependence of color change behavior in *G. aculeatus* in more detail by examining whether and how it was associated with sex, size, boldness, and *Schistocephalus solidus* infection, a parasite known to manipulate antipredator behavior in *G. aculeatus* (Ness and Foster 1999; Barber et al. 2004) and to impair somatic energy stores (Schultz et al. 2006). The use of a repeated design in which individuals were sequentially exposed to the same background twice, allowed us to quantify individual consistency in color change expression. In the second manipulative experiment, we evaluated whether and how the color change behavior was affected by predation risk. If the variation in color changing behavior involves genetic components and is of adaptive value to individuals, we predicted that the degree of color change should 1) vary between species, 2) vary among but be consistent within individuals from the same population, 3) be more strongly expressed in the perceived presence of predators, and 4) be reduced in *S. solidus* infected individuals.

## METHODS

### Sampling and husbandry of fish prior to color change experiments

Focal individuals (subjects) representing both species of sticklebacks (*N*_*G. aculeatus*_ = 117, *N*_*P. pungitius*_ = 9) were collected simultaneously at the 1 March 2017, in the south-western coastal Baltic Sea (56°05.733′N 15°51.449′E, Torhamn, Sweden) using a cast-net (diameter 2.2 m, mesh size 8 mm) and immediately transported to the laboratory facility at the Linnaeus University, Kalmar, Sweden. To standardize pre-experiment conditions and avoid sexual maturation, subjects were initially stored in a 450-L holding tank equipped with a flow-through system of filtered brackish water directly from the Baltic Sea (7 PSU, heated to ∼13 °C) and with a 12L:12D photoperiod. No individual expressed nuptial coloration or breeding behavior during the experiments. Acclimatization in the holding tank lasted for 3–5 weeks (depending on when individuals were assayed for color change capacity) with subjects being fed daily ad libitum with Chironomidae larvae (Akvarieteknik, Sweden).

To reduce the potential effects of social interactions and to standardize the level of satiation, subjects were transferred to 0.8-L opaque gray plastic cups (Hammarplast, Sweden) filled with filtered brackish water and kept in isolation without food for 48 h prior to the color change assay. Water temperature in the bins were kept at 14.7 °C ± 0.4 SD, with a light intensity estimated at 10–20 LUX (Hagner, Digital Luxmeter EC1) and a 12L:12D photoperiod resembling natural conditions at the time of sampling.

### Experiment 1—Assessing sources of variation in color changing behavior

To quantify and examine sources of variation in color change behavior, subjects (*G. aculeatus*, *N* = 69; *P. pungitius*, *N* = 9) were placed in a test arena (28 × 28 × 16 cm transparent glass aquaria filled to 5.5 cm/4.3 L with filtered brackish water 14.7 °C ± 0.4 SD and washed thoroughly after each assayed individual (to avoid olfactory cues to be transferred between assays). Subjects were exposed to dark (black) and light (white) background treatments by manually placing laminated screens of each background treatment along the sides of the aquaria (dark screen: 9 L*; light screen: 157 L*, see below for colorimetric units). Changing between these backgrounds took less than 10 s to perform. Background treatments were chosen to expose individuals to two extreme situations along a dark-light continuum, thereby encouraging subjects to maximize their color changing behavior in both directions (flexibility, the ability to become both darker and lighter). It was also chosen to act as a proxy of variable backgrounds such as light and dark bottom substrates and vegetation as well as variation in light conditions due to turbidity, coloration, and light penetration (Seehausen et al. 1997; Monteith et al. 2007). The bottom of the test arena had medium gray color (lightness: 71 L*) in all trials to allow a more accurate and robust quantification of fish color unaffected by the type of background treatment (see below).

**Figure 1:**
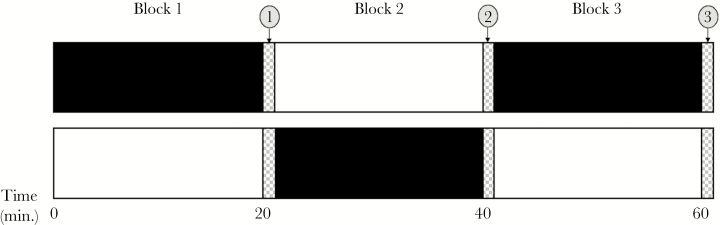
Experimental design to examine individual variation in the behavior to change color in response to a changing background. Subjects were exposed to two backgrounds, dark and light, during three 20-min blocks. The sequence was alternated among subjects such that 50% of the subjects experienced dark–light–dark and the other 50% light–dark–light. The checkered areas denote time points when the background was lifted and frame captures for image analysis were taken without any backgrounds present (clear sides). This was done to eliminate any effects the backgrounds may have on the perceived color of the fish. The three treatments of the predation-risk experiment (control, simulated attack, and olfactory cues) were introduced at time point 2.

Each individual assay of color change lasted 60 min and comprised of three sequential 20-min blocks of exposure to either dark and light backgrounds. To be able to assess repeatability for repeated exposure to both the dark and the light backgrounds, we alternated the initial background and order of treatments (D:L:D or L:D:L, time points 1 and 3; Figure 1). The exposure time of 20 min per background was based on previous findings by Clarke and Schluter (2011) that 15 min is adequate for complete physiological color change in *G. aculeatus* and we confirmed this by conducting pilot trials.

To minimize visual disturbances during color change assays, the test arena was visually sealed off from the rest of the laboratory. The arena was lit using two pairs of fluorescent lamps (Osram L 18W/840 Lumilux, Cool White) placed 1.4 m above the water. This created a uniform lighting within the test arena (255 lux) and minimized glares in the water surface.

### Experiment 2—Evaluating color change in response to perceived predation risk

To evaluate whether and how a perceived predation risk, mediated through simulated attacks or predatory olfactory cues, influenced color changing behavior in *G. aculeatus*, we used an experimental design similar to the one above. However, all these subjects (*N* = 45) were exposed to changing backgrounds in the order dark–light–dark, as this yielded a higher repeatability of dorsal coloration (see Results). During the final change of background (from light to dark; Figure 1, time point 2), subjects were exposed to one of three, randomly allocated, treatments; 1) *control*; adding 0.3 L of regular tank water to the aquarium; 2) *predatory attack simulation*; adding 0.3 L of tank water combined with a simulated predation attack by chasing the individual with a dip-net (10 × 10 cm) for 5 s, which was repeated every 5 min; and 3) *predator olfactory cues*; adding 0.3 L of tank water containing olfactory cues (see below for details) of pike (*Esox lucius*), a natural predator to *G. aculeatus* in the Baltic Sea (Jacobson et al. 2019; Nilsson et al. 2019). Following each individual assay, the aquaria were carefully washed and dried three times to make sure that all olfactory cues of *E. lucius* and subjects were removed. By comparing time points 1 and 3 (Figure 1), this experiment allowed us to assess the effect of a perceived predation risk on the degree of dorsal darkening in the response to a dark background. In addition, it allowed us to examine whether any individual increased response due to perceived predation risk was associated with their “baseline” color change, that is their color change in the absence of predation risk (time points 1 and 2; Figure 1).

Olfactory cues of *E. lucius* were generated similarly to the procedure described by Brönmark and Pettersson (1994). For this purpose, we collected five *E. lucius* (48.5 cm ± 5.6 SD) at Lerviksbäcken (57°04.385′N 16°31.334′E) using a fyke-net (for more details on the method and location, see Tibblin et al. 2016, 2015). These cue-donor individuals were kept at the laboratory in a large holding tank (80 × 140 × 40 cm, 450 L) with artificial vegetation and a flow-through system continually providing the aquarium with brackish water directly from the sea. To generate water containing *E. lucius* olfactory cues, we randomly transferred three individuals to a holding aquarium (40 × 80 × 36 cm, 115 L) filled with water of the same temperature as in Experiment 2 (14.7 °C ± 0.4 SD). After 2 h in the holding aquarium, the *E. lucius* were transferred back to the larger tank. The water was subsequently used as the olfactory cue treatment in trials conducted the same day.

### Real-time recording and frame selection

It is challenging to accurately quantify color change in fishes with photography or spectrometry, as any handling of individuals can bias the results (Stevens et al. 2007; Troscianko and Stevens 2015; Stevens 2016). We therefore chose to record all color change assays with a 4k Ultra-high definition video camera (SONY FDRAX33). It was mounted 50 cm above the center of the test arena, exposure was locked to F:1.8 and 1/50 s, and focus and white balance were set manually prior to each recording. From the video recordings, 8.3-megapixel frame-grabs were taken to quantify the dorsal coloration of the sticklebacks. We selected frames with minimal motion blur and glares from the 10-s windows (time points 1–3) between backgrounds (Figure 1).

### Quantifying color change using image analysis

The dorsal coloration of subjects was quantified by pixel analysis of the full dorsal region in Adobe Photoshop (version CC 2017.0.1). The dorsal area of the subjects was selected with the lasso tool, and the mean dorsal lightness was used as a measurement of dorsal coloration. For lightness we used the L* channel of the “Lab color space” in Adobe Photoshop (version CC 2017.0.1). This color space is equivalent to the frequently used International Lighting Commission Lab color space (CIE; Chen and Hao 2004; Svensson and Nilsson Sköld 2011). In Photoshop, L* ranges from 0 (black) to 255 (white). To compensate for minute differences in the evenness of the lightning, the L* values were adjusted according to the position of the subjects in the arena (Supplementary Figure S1). The CIE color system is based on the human visual system; however, as this study focuses on changes in the lightness of the individual, estimated changes should also be expected to transfer to organisms with a different visual system (i.e., fish and birds).

### Boldness assay

Immediately following the color change assays in Experiment 1, *G. aculeatus* subjects were transferred, using a dip-net, to a refuge box (17 × 17 × 27.5 cm) placed inside a glass aquarium (60 × 30 cm, filled with 23 L) to be assayed for boldness. After an acclimatization period of 20 min in the refuge box, a door was remotely opened to allow subjects to freely explore the aquarium. The time taken to fully emerge from the refuge box was used to calculate boldness. Individuals that had not excited the box within 40 min were assigned to this ceiling value (Nordahl et al. 2018). Refuge emergence time was converted to a boldness score (*B*) according to *B* = 1 − (*t*/40) where *t* is the time taken (in minutes) until the subject emerged from the refuge box, and 40 is the ceiling value.

### Determination of length, sex, and parasite load

Following the behavioral trials in Experiment 1, subjects were individually housed and fed in 4-L cages until the termination of the experiment when they were euthanized by an overdose of benzocaine. We measured total length (to the closest mm) on all subjects and performed dissections to determine sex (gonadal inspection) and the presence of any *S. solidus* parasites (Barber et al. 2004). All *S. solidus* were larger than 50 mg, that is, they had reached a size where they can infect birds. Individuals assayed for color change relating to predation risk (Experiment 2) were kept alive to be included in a breeding program, and thus, were not assessed for additional phenotypic dimensions.

### Ethical approval

The study was carried out in accordance with all relevant applicable national guidelines for the care and use of animals. Ethical approval for this study was granted by the Ethical Committee on Animal Research in Linköping, Sweden (approvals Dnr 52-10 and Dnr 93-15).

### Statistical analyses

First, we tested whether the degree of color change, estimated as the within-individual difference in dorsal lightness when sequentially exposed to dark and light backgrounds (time points 2 and 3; Figure 1) differed between the two species (*G. aculeatus*, *N* = 69 and *P. pungitius*, *N* = 9) using Welch’s *t*-test due to unequal variances. Next, we evaluated whether the degree of color change in response to dark versus light backgrounds was positively related in *G. aculeatus* individuals. To this end, we performed a Pearson correlation with the color change (∆*L*) during the first background as predictor and change during the second background as response (i.e., comparing L* at time points 1 vs. 2 and 2 vs. 3; Figure 1).

To examine whether the expression of color change varied among but was consistent within *G. aculeatus* individuals (*N* = 69), we estimated trait repeatabilities using the intraclass correlation coefficient (ICC). Half of the subjects were exposed to a dark background twice (*N* = 35), whereas the others (*N* = 34) were exposed to a light background twice. Therefore, repeatability was calculated separately for dark and light backgrounds by quantifying dorsal lightness (L*) at time points 1 and 3 (Figure 1). Repeatability estimates were obtained using one-way Anovas as SS_B_^2^/(SS_B_^2^ + SS_W_^2^), where SS_B_^2^ = (MS_B_ − MS_W_)/*k*, SS_W_^2^ = MS_W_, *k* = number of measurements per individual (= 2), and MS_W_ and MS_B_ are the within and between individual mean squares, respectively (Boake 1989; Wolak et al. 2012).

Next, to evaluate potential sources of variation in color changing behavior, we assessed whether the magnitude of color change (time points 2 vs. 3; Figure 1) in *G. aculeatus* (*N* = 69) was associated with other phenotypical dimensions represented by body size (total length mm; range 40–73 mm), sex (37 females; 23 males; 8 n/a), boldness, and presence/absence of parasite infection (42 uninfected; 24 infected; 3 n/a). To this end, data was analyzed by General Linear Models (family = Gaussian, link = identity) with sex and parasite infection as categorical, and body size and boldness as continuous variables. A full factorial model was simplified by stepwise elimination of the highest order nonsignificant (*P* > 0.05) interactions (Crawley 2013) (Table 1). This final model included significant interactions between parasite infection and boldness (*P* = 0.014), and parasite infection and sex (*P* = 0.011). We therefore reanalyzed the data separately for infected (*N* = 21) and uninfected (*N* = 39) individuals, using the same statistical approach as above, which resulted in final models without any interactions (Table 1).

**Table 1:** Model selection and parameter estimates in analyzing color change expression (experiment 1). Reported are coefficients (estimate), SEs, and *P*-values from all statistical models. Bold terms were included in the final, minimal adequate model. Terms in italics were dropped from the final model during model simplification and are displayed with the estimates and probabilities when last included in the model

Parameters	Estimate	SE	*P*
Color change, full dataset			
** Intercept**	23.68	6.80	0.001
** Sex**	−3.99	1.89	0.039
** Length**	−0.038	0.11	0.72
** Parasite infection**	−13.30	4.70	0.007
** Boldness**	−7.54	−2.11	0.039
** Sex × Parasite infection**	8.02	3.03	0.011
** Parasite infection × boldness**	13.79	5.45	0.014
** ** *Sex* × *Length* × *Parasite infection* × *Boldness*			*0.92*
** ** *Sex* × *Length* × *Boldness*			*0.99*
** ** *Sex* × *Parasite infection* × *Boldness*			*0.78*
** ** *Length* × *Boldness* × *Parasite infection*			*0.29*
** ** *Sex* × *Length* × *Parasite infection*			*0.22*
** ** *Sex* × *Length*			*0.78*
** ** *Sex* × *Boldness*			*0.77*
** ** *Length* × *Parasite infection*			*0.70*
** ** *Length* × *Boldness*			*0.64*
Color change, uninfected individuals			
** Intercept**	26.30	8.92	0.006
** Sex**	−4.12	1.81	0.03
** Length**	−0.08	0.15	0.57
** Boldness**	−7.66	3.38	0.03
** ** *Sex* × *Length* × *Boldness*			*0.96*
** ** *Length* × *Boldness*			*0.52*
** ** *Sex* × *Boldness*			*0.50*
** ** *Sex* × *Length*			*0.37*
Color change, infected individuals			
** Intercept**	*7.86*	*11.25*	*0.49*
** Sex**	*4.14*	2.75	*0.15*
** Length**	*0.00*	*0.16*	*0.99*
** Boldness**	*6.45*	*4.73*	*0.19*
** ** *Sex* × *Length* × *Boldness*			*0.95*
** ** *Sex* × *Boldness*			*0.86*
** ** *Length* × *Boldness*			*0.53*
** ** *Sex* × *Length*			*0.36*

To evaluate the effects of perceived predation risk on color change, we used general linear models. Additional color change, that is, the difference in the degree of color change before and after treatment, was used as response variable, and the full factorial model included the factors of treatment and color change prior to treatment (i.e., time point 1 vs. 2; Figure 1). Removal of nonsignificant interactions (simulated attack × initial color change; *P* = 0.935; olfactory cue × initial color change, *P* = 0.682) resulted in a final model without interactions. To further investigate the relationship between the response to perceived predation risk (control treatment excluded) and initial degree of color change, we also performed a regression analysis with initial change as predictor and additional change as response with data from both treatments pooled.

All statistical analyses were conducted in RStudio v.1.163, with R v.3.5.2 (R Core Team 2016) using the packages Psych v. 1.7.5 (ICC analysis) and Stats v. 3.5.2 (GLMs). Assumptions of all GLMs were checked by visual inspections of the residuals in diagnostic plots with little deviations in terms of normal distribution and equal variances (Crawley 2013).

## RESULTS

### Degree of color change between species and consistency within individuals

**Figure 2:**
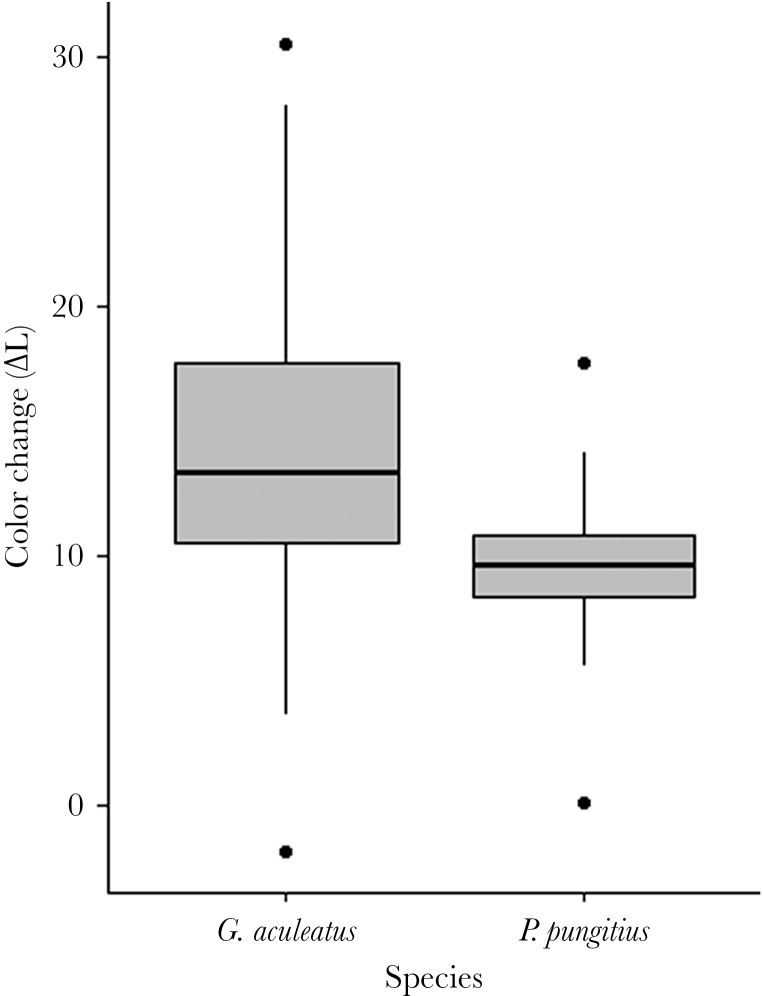
Comparison of the degree of color change between *Gasterosteus aculeatus* and *Pungitius pungitius* individuals experimentally exposed to dark and light visual backgrounds. A large color change value (ΔL*) indicates a large difference in dorsal color between the dark and light background, and a zero indicates that no change in coloration (L*) occurred. The boxes indicate medians, 25th and 75th percentiles, and the whiskers below and above indicate the 5th and 95th percentiles.

**Figure 3:**
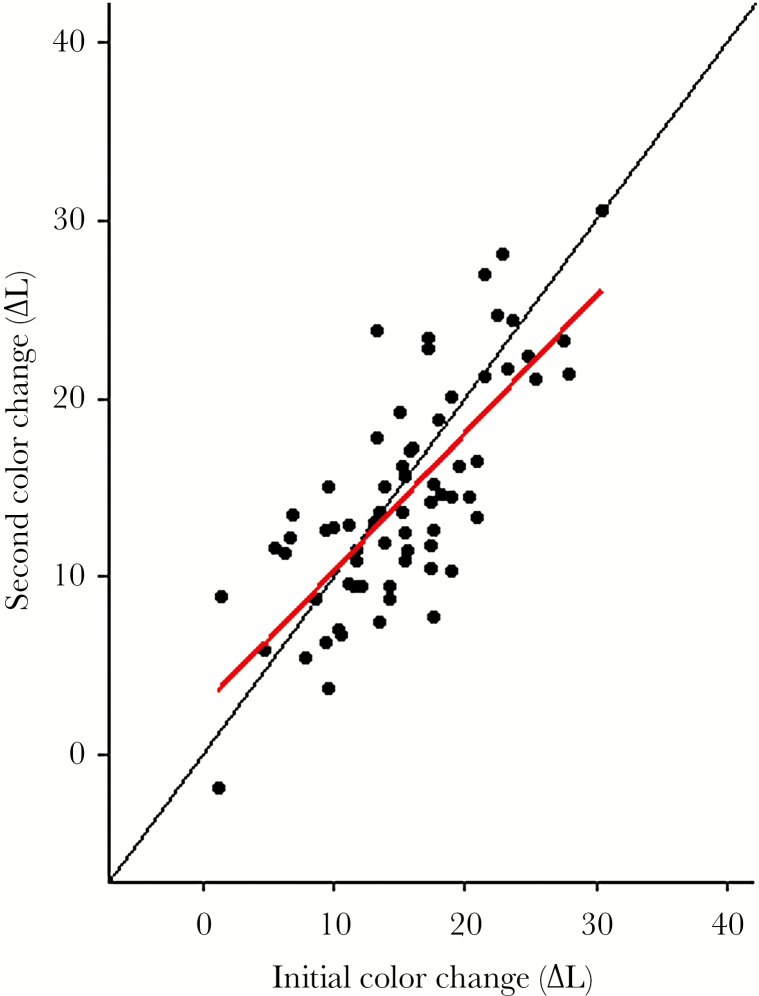
The relationship between the degree of individual color change of *Gasterosteus aculeatus* (*N* = 69) when exposed to sequential changes in visual backgrounds, from light to dark or vice versa. The black and red lines denote the 1:1 ratio and linear regression, respectively.

When the visual backgrounds were changed, *G. aculeatus* changed their dorsal coloration more than *P. pungitius* (mean ± SD of ∆L color change: *G. aculeatus* = 14.38 ± 6.24 SD., *P. pungitius* = 9.44 ± 4.99 SD; Welch’s *t*-test, *t*_11.54_ = 2.71, *P* = 0.02; Figure 2). In *G. aculeatus*, the degree of color change that was expressed when exposed to dark versus light backgrounds were closely related (Pearson correlation, *r* = 0.75, df = 67, *t* = 9.30, *P* < 0.001; Figure 3). This means that individuals were consistent in their color change behavior such that individuals showing a strong response to the introduction of a dark background also showed a strong response to the light background, whereas individuals that changed little did so in both situations. This pattern of individual consistency in color change was also found when estimating repeatability in coloration of *G. aculeatus* that were exposed to the same background twice. That is, individual *G. aculeatus* consistently expressed similar dorsal coloration (L*) following repeated exposures to dark, or to light, backgrounds. Repeatability estimates were statistically significant and of comparable magnitude for the dark background (*R* = 0.80, *P* < 0.001, *N* = 35) and for the light (*R* = 0.51, *P* < 0.001, *N* = 34) background.

### Associations of color change with other phenotypic dimensions

**Figure 4:**
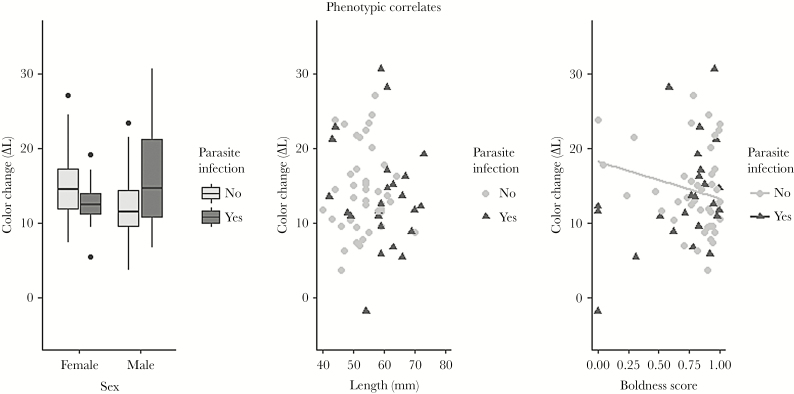
Associations of color change with parasite infection, sex, body size, and boldness in *Gasterosteus aculeatus*. Data are presented separately for *S. solidus* parasite infected (light gray) and uninfected individuals (dark gray) due to significant interactions effects (see Results).

The associations of phenotypical traits with the color changing behavior depended on whether individuals were infected by the *S. solidus* parasite, as evident by significant interactions between infection and boldness, and infection and sex (GLM; infection × boldness: *t*_53_ = 2.58, *P* = 0.01; infection × sex: *t*_53_ = 2.66, *P* = 0.01). In uninfected individuals, males changed color less than females (mean ∆*L* color change: males = 12.45 ± 5.46 SD, females = 15.39 ± 5.25 SD; GLM, effect of sex; *t*_35_ = 2.28, *P* = 0.03), and bolder individuals changed color to a lesser degree (GLM, effect of boldness; *t*_35_ = 2.27, *P* = 0.03; Figure 4). In infected individuals, color change was not associated with sex or boldness (GLMs; sex: *t*_17_ = 1.51, *P* = 0.15; boldness: *t*_17_ = 1.36, *P* = 0.19; Figure 4). Color change was unaffected by body size in both infected and uninfected individuals (GLMs total length; infected: *t*_17_ = 0.003, *P* = 0.99; uninfected *t*_35_ = −0.57, *P* = 0.57; Figure 4).

### Predation risk influence the expression of color change

**Figure 5:**
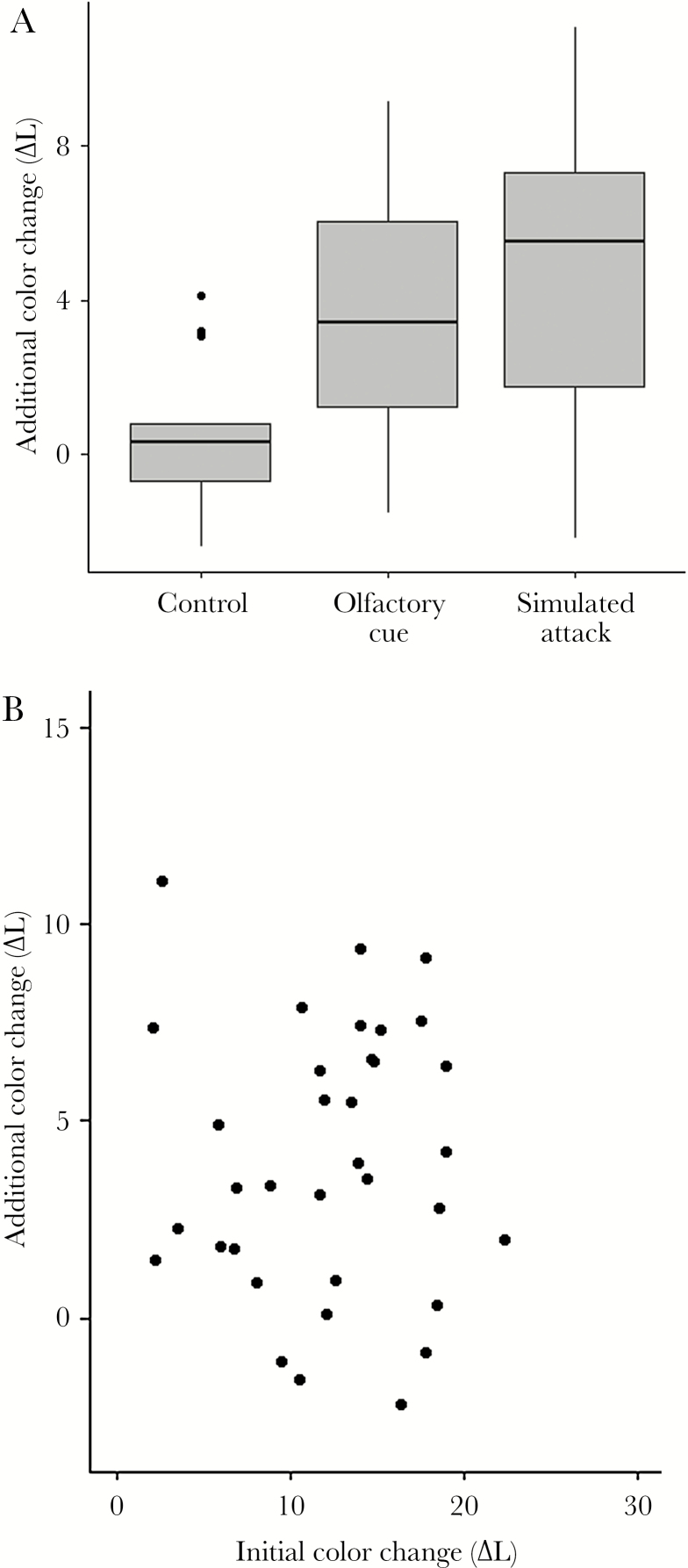
Effects of predation risk on the expression of color change in *Gasterosteus aculeatus*. (a) Additional expressions of color change in control individuals, and individuals exposed to predator olfactory cues or simulated predator attack (stress) treatments. (b) Individual associations between initial (pretreatment) and additional (post simulated predation risk treatment) expression of color change. Data represent responses to olfactory cues and simulated predator attacks.

Subjects were first exposed to a dark background without predator cues and the resulting change in coloration was compared with that following a second exposure to a dark background, this time with including predator cues (as well as a control). Individuals that were experimentally exposed to a simulated predation risk (simulated attack or olfactory cue) responded by increasing their expression of color change in relation to the control treatment (mean additional color change: olfactory cues = 3.60 ± 3.06 SD, simulated attack = 4.37 ± 3.74 SD, control = 0.59 ± 1.87 SD; GLM; effect of olfactory cue: *t*_41_ = 2.09, *P* = 0.04; effect of simulated attack: *t*_41_ = 2.76, *P* = 0.01; initial color change: *t*_41_ = 0.24, *P* = 0.81 Figure 5a). The extent of the additional color change elicited by the two predation treatments was independent of the initial color change prior to treatment (regression analysis, *F*_1, 33_ = 0.002, *P* = 0.96; Figure 5b).

## DISCUSSION

In this study, we explored individual variation in the expression of reversible rapid color change in two closely related model fish species in ecology and evolution, *G. aculeatus* and *P. pungitius*. We quantified individual expression of color change in response to a changing visual environment using digital image analysis of high-resolution video recordings. Our results show that alternating backgrounds induced changes in the dorsal coloration, and that this response: 1) differed between species, 2) varied among but was highly consistent within individuals, even though color change was expressed in opposite direction to match both darker and paler backgrounds, 3) was not affected by body size, but 4) were affected by sex and boldness in individuals not infected by *S. solidus*. Finally, we show that 5) the color changing response was enhanced under perceived predation risk but independent of the baseline color change in the absence of predator cues.

Our study adds to the current body of knowledge that there is substantial intrapopulation variation in phenotypic flexibility (Nussey et al. 2007; Kang et al. 2016). We found that individuals differed in their color change response to background treatments representing the extremes along the light–dark continuum and that the magnitude of the responses to darker and lighter visual backgrounds were correlated. What, then, are the underlying sources of this variation in flexibility among individuals? By investigating associations with multiple phenotypical dimensions, we showed that color changing behavior was independent of body size but varied according to sex and boldness, such that males and bolder individuals changed color to a lesser extent. The lack of association between body size, which is also a proxy for age, and color change suggest that previous experience and ontogeny had little impact on color changing behavior (DeFaveri and Merilä 2013; Senner et al. 2015). This may point toward a genetic basis to individual variation in color change behavior although formal evaluation of this would require either quantitative genetic or genomic studies. Regarding the patterns with sex and boldness, this may be reflective of the potential costs associated with color change. Male *G. aculeatus* have an impressive capacity to express chromatic nuptial coloration (red–green–blue) that may come at the cost of less efficient color change along the dark–light continuum (Milinski and Bakker 1990; Brock et al. 2017). For boldness, theory and empirical evidence suggest that bolder individuals are likely to be more vulnerable to predation, and it has been hypothesized that boldness should be positively correlated with antipredator traits (Hulthén et al. 2014) such as color change capacity (Stevens 2016; Duarte et al. 2017). However, our results indicated instead that the degree of color change was less—not more—pronounced in bolder individuals, perhaps indicating that they were less prone to conceal themselves in novel environments (Fraser et al. 2001; Mazué et al. 2015).

Our results also suggested that the associations of color change with sex and boldness were modified by *S. solidus* parasite infection, but contrary to our prediction, the degree of color change was not reduced in infected individuals in general. As these wild-captured individuals were naturally infected, one needs to be careful regarding the causality of this pattern. It is known that melanin-based coloration, which is involved in the darkening of dorsal regions, can influence the outcome of interactions with parasites such that high melanin content is associated with higher parasite resistance (Côte et al. 2018). On the other hand, the *S. solidus* parasite can manipulate *G. aculeatus* to reduce its antipredator behavior (Ness and Foster 1999; Demandt et al. 2018) and also impair nuptial color change (Milinski and Bakker 1990; Candolin and Voigt 2001). Moreover, many parasites, including *S. solidus*, commonly manipulate multiple phenotypic dimensions of their host, which may result in complex interactions as demonstrated in this study (Thomas et al. 2010). Nonetheless, the present study provides rare evidence on that the link between rapid color changing behavior and other phenotypic dimensions may be modulated by parasite infection (but see Milinski and Bakker 1990; Skarstein and Folstad 1996; Candolin and Voigt 2001 for related examples).

In addition to substantial intrapopulation variation in the degree of color change, our results also revealed that individuals consistently changed their color so that their responses to different visual backgrounds were strongly correlated. The latter was further supported by a high repeatability in the color that individuals expressed when repeatedly exposed to the same background. Consistency (repeatability) has previously been studied in various flexible traits, such as boldness, aggression, and migratory timing, and the results suggest that these traits can be both predictable and heritable (Bakker 1986; Bell et al. 2009; Dingemanse et al. 2010; Tibblin et al. 2016). Our study provides important insights about consistency in the expression of phenotypic flexibility (degree of color change) rather than in coloration itself (Forsman 2015). Admittedly, our estimates only represent short-term consistency, but nonetheless, the results suggest that the degree of color change may involve a heritable genetic component (Boake 1989; Bell et al. 2009). This conclusion is further supported by our interspecific comparison, showing that *G. aculeatus* change color to a greater extent than *P. pungitius*. However, this comparison needs to be interpreted cautiously based on the low n-values of *P. pungitius* and whether these differences are reflective of adaptive divergent evolution remains to be investigated, for instance by combining manipulative experiments with genomic (Momigliano et al. 2017) or quantitative genetic approaches (Leinonen et al. 2013). Likewise, given our findings and that an adaptive genetic basis for differences in color change between two ecotypes of *G. aculeatus* has previously been proposed by Clarke and Schluter (2011), further quantitative genetic studies involving populations differing in their predation regimes and visual environments would provide interesting further avenue to study the genetic and adaptive basis of phenotypic flexibility in color change.

An obvious advantage of physiological color change is the ability to visually match a heterogenous background (Merilaita et al. 1999). This aspect has received extensive scientific attention with the general consensus being that it can decrease detection and reduce predation risk (Endler 1988; Stevens and Merilaita 2009; Endler and Mappes 2017). The threat of predation can induce irreversible plastic antipredator responses (Brönmark and Miner 1992; Ahlgren et al. 2015). However, whether predation risk also induces adjustments of color changing behavior is comparatively less studied (but see Candolin 1998; Garcia and Sih 2003; Stuart-Fox et al. 2008; Edelaar et al. 2017 for related examples). We show that *G. aculeatus* significantly increased the level of color change in response to a perceived predation risk, regardless of whether they were exposed to olfactory cues or a simulated attack. Moreover, the magnitude of this increase in response was independent of the baseline response to a changing background. Similar links between background-mediated color change and predation risk have been reported in crabs (Hemmi et al. 2006), salamanders (Garcia and Sih 2003), grasshoppers (Edelaar et al. 2017), and chameleons (Stuart-Fox et al. 2008), but we believe our study represents the first evidence for this in a fish. Our results contribute novel insights on the modulation of color change and suggest that there is some cost associated with changing color and that fish may adaptively reduce predation risk by increased color change expression.

Our study shows that there was profound interindividual variation in the ability to change color and suggests that this variation may have an adaptive genetic basis. If so, one key question is how this variation can be maintained. The ability to express phenotypic flexibility presumably comes with a cost (Relyea 2002), in the case of color change likely in the form of physiological costs associated with rearrangement of melanin pigments (Stuart-Fox and Moussalli 2009; Stevens 2016). Theory posits that phenotypic and genetic variation can be upheld by fluctuating and opposing selection pressures across the life cycle due to spatial heterogeneity (Hedrick et al. 1976; Roff 1992; Wennersten and Forsman 2012) so that the costs and benefits of high capacity for color change varies spatially. The Baltic Sea *G. aculeatus* migrates between offshore pelagic forage habitats and coastal habitats for reproduction and juvenile life stages whereas *P. pungitius* have a more sedentary life cycle with less distinct offshore migrations (Borg 1985; Bergström et al. 2015). The coastal and offshore habitats differ considerably in habitat heterogeneity, light penetration due to water visibility, and predators, which should influence the interaction between predators and prey (Casini et al. 2009; Reusch et al. 2018; Nilsson et al. 2019). The variation in ability to change color both between species and among individuals within species indicated by our findings may thus be attributable to that spatiotemporal variation in habitat use influences eco-evolutionary dynamics and solutions to variable selection pressures. The characteristics of these habitats are changing rapidly due to ongoing climate change and anthropogenic impacts such as light pollution (Depledge et al. 2010), eutrophication (Seehausen et al. 1997), brownification (Monteith et al. 2007; van Dorst et al. 2019), and overfishing of the predatory fish (Reusch et al. 2018). All these may influence the adaptive value of color change.

Unlike intraindividual seasonal changes in protective coloration that enable animals to cope with long-term and predictable alterations in environmental conditions and selection pressures (Mills et al. 2018; Zimova et al. 2018), our study instead informs about sources of variation in rapid color changes that may confer fitness benefits in rapidly changing and fine-grained environments. The results provide rare evidence on repeatability, phenotypic correlates, and context-dependent adjustments of color change in response to parasite infection and predation risk, thus illustrating how a suite of multiple interacting factors may contribute to variation in intraindividual phenotypic flexibility. The observed interspecific differences also points to a possible adaptive value and evolvability of rapid color change, but different experimental approaches (Forsman and Appelqvist 1999; Forsman and Merilaita 1999; Karpestam et al. 2018) are necessary to evaluate whether and how color changes influence susceptibility to visual predators. Firm evidence would also require that the ability to express color change is experimentally manipulated to compare predation rates on manipulated and control individuals in temporally changing (or spatially heterogeneous) visual environments—a key future challenge for this nascent research field.

## FUNDING

This work was supported by funds kindly provided by the Kalmar and Växjö’s major Linnaeus Scholarship for research within ecological sustainable development (grant to P.T.) and the Linnaeus University (to P.T., A.S., and A.F.).

We are grateful to H. Hallberg, J. Nilsson, O. Nordahl, J. Sunde, and P. Söderling for assistance in the field and the laboratory and to Prof. P. Larsson for providing laboratory resources. We thank J. DeFaveri for invaluable input regarding the research design. We are grateful to S. Merilaita, O. Nordahl, and three anonymous reviewers for valuable comments that improved the manuscript. P.T., A.F., and J.M. conceived the study. P.T., M.H., A.S., and A.F. designed the experiments. M.H. performed experiments, and M.H. and P.T. analyzed the data. All authors contributed to interpretation of results. P.T. drafted the first manuscript. All authors commented and agreed to the final version of the manuscript.

Conflict of interest: We have no competing interests.

Data accessibility: Analyses reported in this article can be reproduced using the data provided by Tibblin et al. (2020).

## Supplementary Material

araa041_suppl_Supplementary_Material
