# Supplementary material for: Phenotypic flexibility in background-mediated color change in sticklebacks
Source: Behav Ecol. 2020 May 6;31(4):950–9. doi: 10.1093/beheco/araa041 (PMC7390996; doi:10.1093/beheco/araa041)
Supplement: araa041_suppl_Supplementary_Material [file araa041_suppl_supplementary_material.docx]

# Supplementary material - Standardization scheme

To compensate for minute differences in the evenness of the lightning all framegrabs were adjusted according to a standardization scheme. The grey bottom of the test arena was divided into squares. Squares with clear heterogenous shading were further divided into smaller subsections (*i.e.* yellow squares) and analyzed separately. The lightness (L*) in each square was estimated in relation to the lightness value of the center grey square (C3). The lightness (L*) in each square was then adjusted to the lightness (L*) value of the C3 square using the mid-lever in the levels menu for lightness in Adobe Photoshop, until each square had the same lightness (L* value) as the C3 square. The adjustment level used to bring the square to the same L* as center square was used as the standardization scheme. (*i.e.* Figure S.1) . Areas with glare reflections, as well as any ink markings that separated the squares, were excluded in the estimation of L* for the squares. Squares with significant glare (red) was estimated as well but avoided during the selection of framegrabs used for estimating dorsal coloration for subjects.

Each framegrab used to quantify dorsal coloration of subjects were adjusted according to the standardization scheme (Fig. 1) such that the image was lightened or darkened to match the lightness of the C3 baseline. For instance, if the subject in the frame grab was positioned in square D4, the image was darkened slightly by adjusting the mid-lever in the levels menu to 0.97.

*
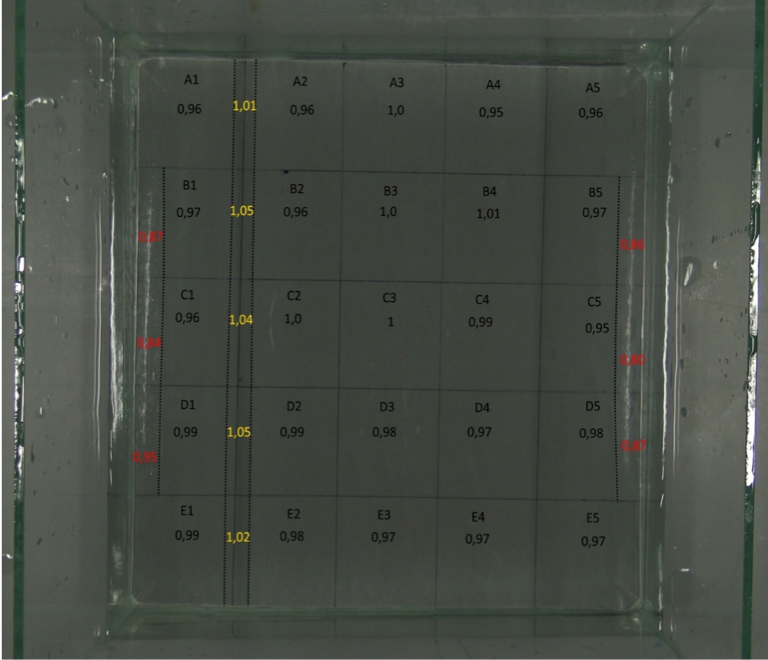
*

Figure S1: Standardization scheme. Lightness in images were standardized in Adobe Photoshop by adjusting the mid-lever in the levels menu for lightness to the values noted in the image above, depending on the position of the subject (i.e. the square occupied at the time of the framegrab).
